# Design of a Compact 2–6 GHz High-Efficiency and High-Gain GaN Power Amplifier

**DOI:** 10.3390/mi15050601

**Published:** 2024-04-29

**Authors:** Yongchun Zhou, Shuai Wang, Junyan Dai, Jiang Luo, Qiang Cheng

**Affiliations:** 1 State Key Laboratory of Millimeter Waves, Southeast University, Nanjing 210096, China; yczhouseu@163.com (Y.Z.); junyand@seu.edu.cn (J.D.); 2 School of Electronic Science and Engineering, Nanjing University, Nanjing 210096, China; wangshuai625@126.com; 3 School of Electronics and Information, Hangzhou Dianzi University, Hangzhou 310018, China

**Keywords:** ultra-wideband, GaN, high efficiency, power amplifier

## Abstract

In this paper, a novel wideband power amplifier (PA) operating in the 2–6 GHz frequency range is presented. The proposed PA design utilizes a combination technique consisting of a distributed equalization technique, multiplexing the power supply network and matching network technique, an LR dissipative structure, and an RC stability network technique to achieve significant bandwidth while maintaining superior gain flatness, high efficiency, high gain, and compact size. For verification, a three-stage PA using the combination technique is designed and implemented in a 0.25 μm GaN high-electron-mobility transistor (HEMT) process. The fabricated prototype demonstrates a saturated output power of 4 W, a power gain of 21 dB, a gain flatness of ±0.6 dB, a power-added efficiency of 39–46%, and a fractional bandwidth of 100% under the operating conditions of drain voltage 28 V (continuous wave) and gate voltage −2.6 V. Moreover, the chip occupies a compact size of only 2.51 mm × 1.97 mm.

## 1. Introduction

Monolithic microwave integrated circuit (MMIC) power amplifiers play a vital role in RF transceiver modules for radar systems, communication systems, and satellite systems, and the performance of the entire system is closely related to the bandwidth, output power, efficiency, reliability, and other indicators of the power amplifier. In recent years, compact broadband high-efficiency MMIC power amplifiers have been widely used, making their design a research hotspot. Compared to other semiconductor processes such as GaAs and SiGe, GaN technology offers higher electron saturation velocity, higher breakdown voltage, and a wider bandgap, making it exceptionally well suited for achieving broadband high-efficiency PAs [1,2,3,4].

Most of the reported broadband high-efficiency PAs currently adopt balanced and distributed circuit architectures [5,6,7]. While these designs achieve wideband performance and excellent return loss characteristics, they often sacrifice efficiency and require larger chip sizes, thereby limiting their widespread application in high-efficiency, low-cost systems. In reference [8], a 2–6 GHz 10 W GaN PA MMIC was introduced, employing an output matching technique utilizing drain shunt capacitors with tapered capacitance to minimize chip area and enhance input and output return loss. However, the PA exhibits only 27% to 34% efficiency across the entire frequency band. Reference [9] presents a design approach for broadband PAs merged with a minimum-inductance bandpass filter (BPF) network, which can provide higher out-of-band attenuation and lower in-band insertion loss. Nevertheless, the PA exhibits a large output power variation of 35.1–38.9 dBm over a bandwidth of 2–4 GHz. A multiresonance harmonic matching technique was employed for the continuous class-F-mode operation to support a broad bandwidth and obtain a compact chip area [10]. The insertion loss of the output matching network was 0.6–1.1 dB over the bandwidth of 4–6 GHz, leading to higher power consumption. In order to achieve gain flatness, a good bandwidth, efficiency, and linearity, Xuan et al. reported an LRC equalizer that flattened the small-signal gain and a four-way zero-degree combiner that matched the fundamental and second harmonic impedances [11]; an MMIC PA operating from 2 to 6.5 GHz with the mentioned techniques was fabricated. Since the loss of the combiner increases so fast at the high-frequency end, the output power at 6 GHz decreased by 2 dB from the maximum value, and its efficiency decreased by 17% from the maximum one. Additionally, a broadband GaN HEMT power amplifier based on the feeding capacitance compensation method is described in Reference [12] with a bandwidth of 2.2–5.1 GHz and an output power of 38.5–41.5 dBm. A gain equalization technique was employed in the inter-stage matching circuit and a low-loss output matching network was utilized to ensure high efficiency [13]; however, the size of the chip reached 14.35 mm^2^. A novel topology of coupled resonators was exploited for the broadband inter-stage matching to cover the 802.11ax bands from 2.4 to 6 GHz [14], with amplifiers featuring a gain flatness of about 10 dB over the entire operating frequency band. According to the above investigation, the main challenge in wideband PA design is to tradeoff between bandwidth, gain, flatness, output power, and power-added efficiency. Even though the techniques mentioned above can extend the bandwidth of PAs to some extent, there is still significant demand for effective approaches that can significantly expand bandwidth while maintaining high gain, reasonable flatness, high efficiency, and compact size.

In this paper, we propose an alternative 2 to 6 GHz broadband PA with high efficiency and high gain. By exploiting multiple methods, including the distributed equalization technique, multiplexing the power supply network and matching network technique, an LR dissipative structure, and the RC stability network technique (DE-MPM-LD-RS), the bandwidth of the amplifier is greatly extended with superior large- and small-signal flatness, high efficiency, high gain, and compact size. The proposed design techniques are demonstrated in a three-stage PA using a 0.25 μm GaN HEMT process. The article is organized as follows: Section 2 describes the design of the proposed power amplifier, including general scheme selection and circuit schematic design. Subsequently, a novel wideband PA is designed and fabricated in Section 3. Finally, the paper is concluded in Section 4.

## 2. Proposed Design Method

In order to meet both communication and radar applications, the amplifier is determined to achieve about 4 W (36 dBm) output power, high efficiency, and high linearity. The power density of the 0.25 μm GaN HEMT process is approximately 5.86 W/mm. Typically, the loss of the output stage matching circuit is about 0.5 dB. Therefore, the size of the power transistor in the output stage is determined to be 6 × 125 μm to achieve 36.5 dBm output power. The selection of the static operating point affects the performance of the chip. In this design, the leakage voltage is +28 V, and the static current is selected as 30% of the maximum drain current so that the power amplifier works in Class AB to compromise between efficiency and linearity.

### 2.1. General Scheme Selection

**Figure 1:**
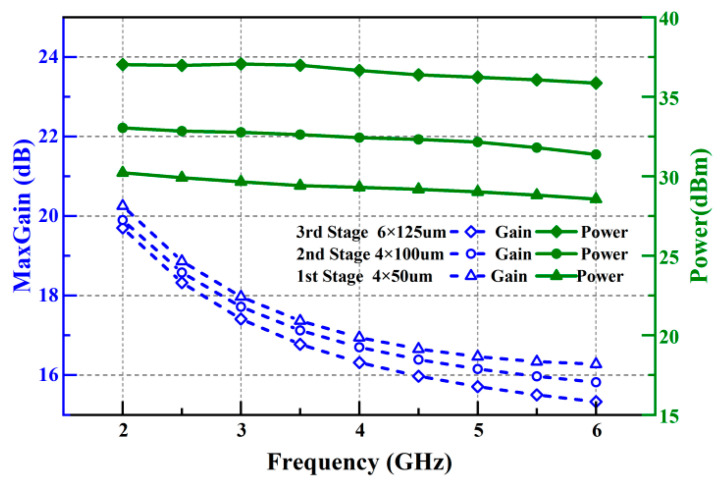
Gain and output power comparison of transistors of different sizes.

Since the output power of a conventional pre-amplifier is typically about 15 dBm and the output power of this PA is about 36 dBm, the power gain of this PA is designed to be 21 dB. To meet the high-gain requirements, this chip adopts a three-stage cascade structure. Figure 1 is a comparison of the gain and output power of transistors of different sizes. It can be seen that the gain of the transistors of smaller sizes is larger, so when the output power of the transistor is enough to drive the next stage, a transistor of a smaller size should be chosen (if possible) to obtain a higher link gain.

**Figure 2:**
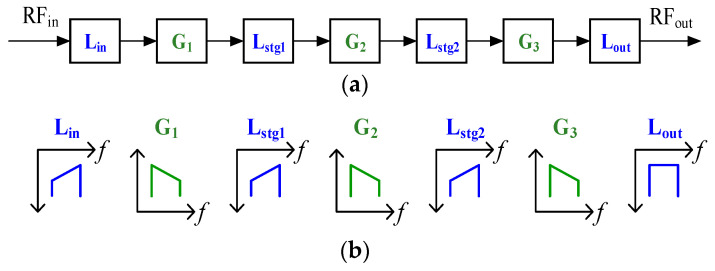
(**a**) The overall structure of the amplifier. (**b**) The gain trend of each stage.

The gain flatness and the power flatness of the broadband power amplifier are important parameters to consider during the design. The bandwidth of this power amplifier reaches triple octave, while the gain of the transistor typically decreases at a rate of 6 dB per octave. Therefore, a suitable equalization structure needs to be selected to achieve excellent in-band gain flatness. In this paper, the equalization of the input matching network and inter-stage matching network are designed to be integrated and multiplexed. To avoid over-equalization at any stage of the circuit, resulting in insufficient gain or power, a distributed equalization technique (DET) is proposed in this paper. The insertion loss of the input matching network and the inter-stage matching networks are, respectively, designed to have a positive slope with frequency, which compensates for the gain and power roll-off characteristics of the neighboring transistors, thus ensuring gain and power flatness throughout the entire frequency band. Figure 2 shows the overall structure of the amplifier (a) and the gain trend of each stage (b). Lin and Lout represent the losses of the input and output matching circuits, Lstg1 and Lstg2 represent the losses of the inter-stage matching circuits, and G1, G2, and G3 represent the gains of the first, second, and third stages of transistors, respectively. Since no equalization is applied to the output matching network and the equalization of the forward matching circuits has little effect on the efficiency of the overall link, better flatness can be obtained using the DET technique without affecting the output power and output efficiency.

### 2.2. Circuit Schematic Design

**Figure 3:**
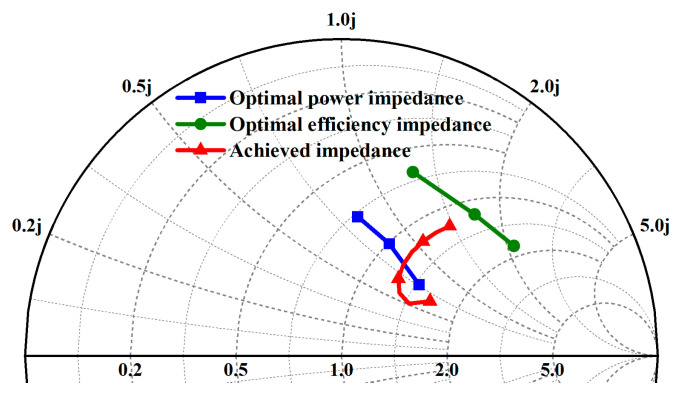
The trend of optimal power, optimal efficiency, and achieved impedance points at different frequencies.

In the frequency range of 2–6 GHz, the optimal power impedance point and optimal efficiency impedance point for the output stage with a 6 × 125 μm transistor are obtained using the load-pull method, as shown in Table 1. Generally, the equivalent efficiency circle and equivalent power circle of the transistor are different circles. For broadband amplifier design, the optimal power impedance point and optimal efficiency impedance point at different frequency points are also different. In this case, we need to balance the output power and efficiency comprehensively. Broadband matching mainly involves impedance matching to ensure power and efficiency at high frequencies while reducing some low-frequency gain. The trends of the optimal power impedance point and the optimal efficiency impedance point on the Smith chart with frequency variation are shown in Figure 3. Based on the analysis mentioned above, the optimal load impedance ZL = (41 + j * 47) Ω is finally selected as the initial value for matching.

**Figure 4:**
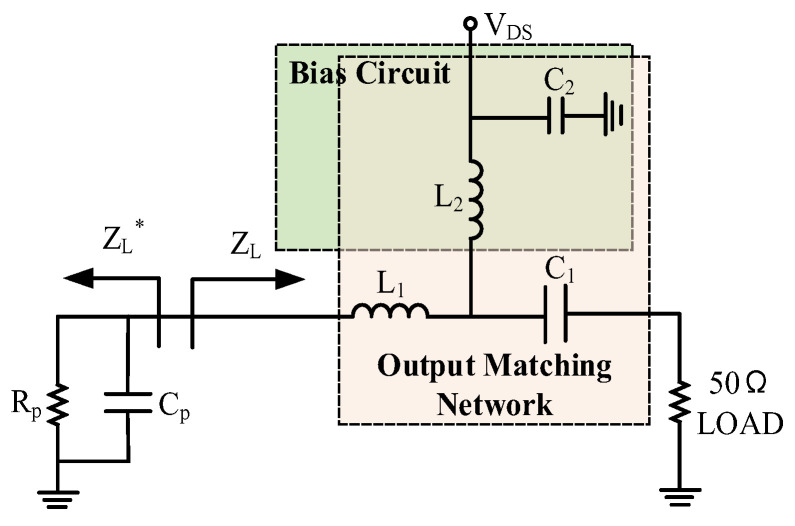
Output matching structure.

The RC model can be equivalent to the large-signal output impedance of the transistor and can be used for the analysis and design of broadband matching networks. According to the impedance transformation relationship shown in Equations (1)–(3), the selected optimal load impedance Z_L_ is equivalent to the RC parallel output impedance model shown in Figure 4. Here, R_p_ and C_p_ represent the equivalent parallel resistance and capacitance in the RC model, γ represents admittance, G represents conductance, B represents susceptance, and ω represents angular frequency. Based on the RC parallel structure, the broadband matching network can be designed.

(1) $$\gamma =\frac{1}{Z_{L}^{*}}=G+jB=0.01+j0.012(S)$$

(2) $$R_{p}=\frac{1}{G}=100(\Omega )$$

(3) $$C_{p}=\frac{B}{\omega }=0.318(pF)$$

**Figure 5:**
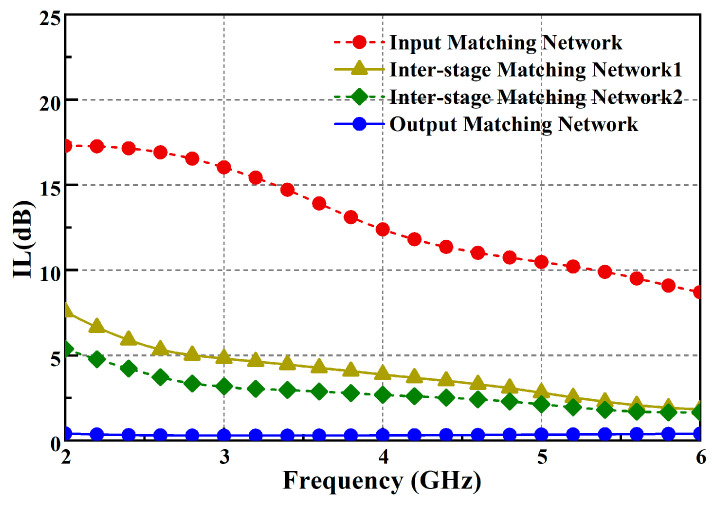
Losses of the matching networks.

The selection of the output matching circuit determines the efficiency and power performance of the amplifier. As shown in Figure 4, the bias circuit uses inductor L2 for feeding and capacitor C2 for decoupling. To minimize the number of components in the output matching circuit to obtain the lowest possible losses, the choke inductor L2 and decoupling capacitor C2 circuits are also included in the matching circuit, generating an inductive impedance that matches the capacitive impedance of the transistor output. In addition, since L2 participates in the output matching circuit, its inductance value is designed to be smaller, and thus its parasitic resistance is smaller, which reduces the DC loss. By multiplexing the power supply network and matching network (MPM), this output matching circuit achieves a very low pass-by loss of only about 0.5 dB after optimization in schematic simulation, as shown in Figure 5. Due to the reduction in the number of components, MPM technology also results in a compact chip size.

**Figure 6:**
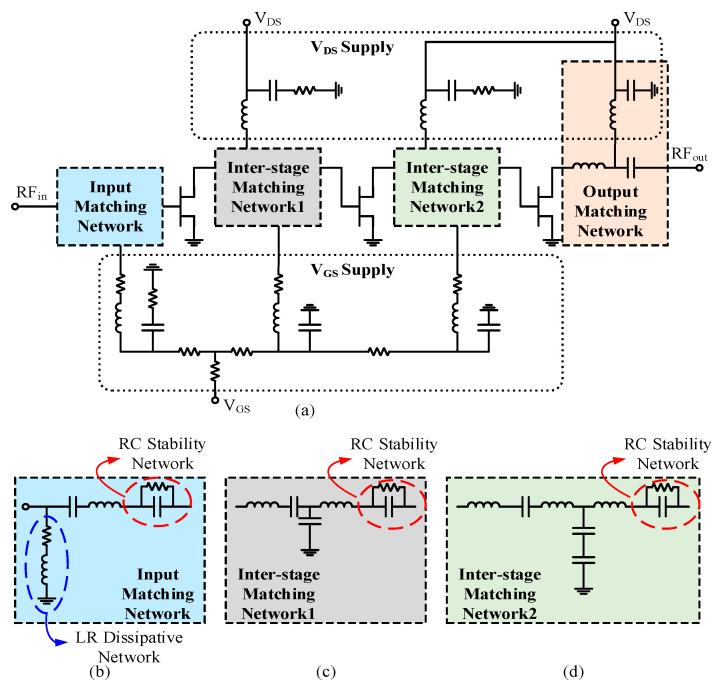
(**a**) Schematic of power amplifier. (**b**) Schematic of input matching network. (**c**) Schematic of inter-stage matching network 1. (**d**) Schematic of inter-stage matching network 2.

The distributed equalization technique (DET) refers to the triple equalization of the inter-stage matching networks and the input matching network. The inter-stage matching networks focus on power matching and match the optimal load impedance of the previous stage transistors with the source impedance of the next stage transistors to ensure power transmission. The design of this network is still based on the aforementioned equivalent RC model and uses a multisection reactance matching network (MRM) to achieve broadband performance, as shown in Figure 6c,d. Gain flatness is mainly taken into account in optimization. As shown in Figure 5, the losses of the inter-stage matching network are high at low frequencies, while at high frequencies, the losses are relatively small and show a positive slope trend to compensate for the negative slope characteristics of the transistors’ own gain.

In addition to the inter-stage matching network, the input matching network structure also needs to consider the performance of gain flatness and input standing wave. In this design, an inductor-resistor (LR) dissipative structure is added to the input, which consists of a resistor and an inductor connected in series and then in parallel to the ground, as shown in the dissipative network circled in Figure 6b, the addition of which can absorb a portion of the low-frequency gain to adjust the gain flatness within the band and also improve the input standing wave performance.

**Figure 7:**
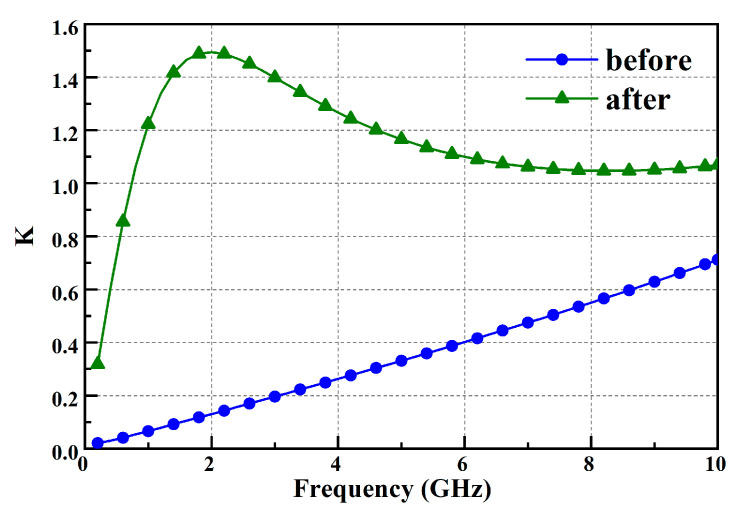
Comparison of stability coefficients before and after joining the stability network.

Amplifiers are prone to instability when operating at high gain and high power. Unstable amplifiers can cause system malfunctions or even damage the system [15]. Therefore, stability indicators must be given special attention during the design process. Due to the inherent instability of transistors within the frequency band, stability networks are added to increase the input impedance of the transistor, as indicated by the circled stable network in Figure 6b–d. This stability network consists of an RC parallel circuit in series with the gate bias circuit of the transistor. Although adding the stability circuit sacrifices about 0.4% efficiency of the amplifier, it can reduce some low-frequency gain and thereby improve the stability of the transistor. The comparison of the stability factor (K-f) curves before and after adding the stability network to the transistor is shown in Figure 7. It can be seen that the transistor is in a stable state after the stability network is added.

## 3. Experimental Validation and Results

**Figure 8:**
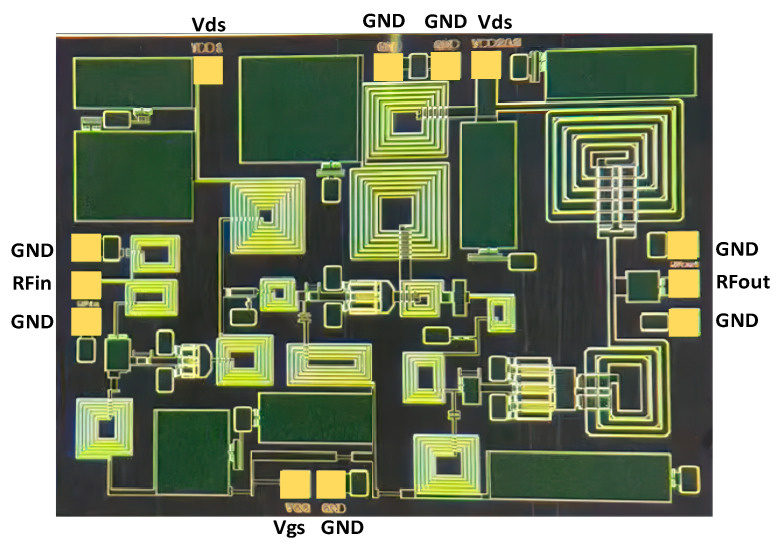
Microphotograph of the PA chip.

The three-stage cascade PA was implemented using a 0.25 μm GaN HEMT process. A die microphotograph of the PA chip is shown in Figure 8, with the whole chip area equal to 2.51 mm × 1.97 mm.

To measure the performance of the PA, the bare amplifier chip was soldered to a Mo-Cu carrier board and the chip was tested on-wafer using a vector network analyzer and a microwave probe station. The chip was tested in the 2–6 GHz frequency range with bias conditions of gate voltage VGS = −2.6 V and drain voltage VDS = 28 V (continuous wave), with an input power Pin = 15 dBm.

**Figure 9:**
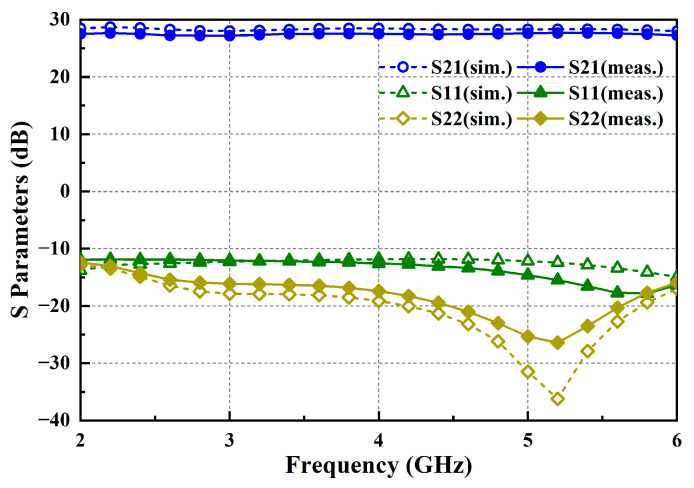
Simulated and measured S-parameters of the PA.

The simulated and measured S-parameters of the proposed PA are plotted in Figure 9, which present good agreement in trend. The measured results indicate that within the operating frequency range of 2–6 GHz, the average linear gain S21 of the amplifier is 27.5 dB, with excellent broadband gain flatness within ±0.6 dB. The input return loss S11 and output return loss S22 are less than −10 dB, respectively.

**Figure 10:**
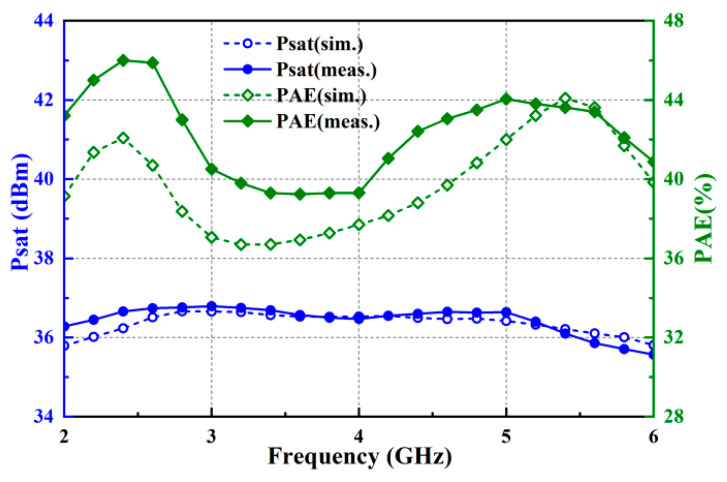
Simulated and measured Psat and PAE.

Figure 10 illustrates the simulated and measured data for the chip’s output power Psat and power-added efficiency PAE. The results indicate that within the frequency range of 2–6 GHz, the output power is 36 dBm, or 4 W, with good power flatness, less than ±0.6 dB, and the power-added efficiency ranges from 39% to 46%. The measured output power aligns well with the simulation curve, while there are some deviations between the measured efficiency and the simulation results. This is mainly due to the difference between the nonlinear simulation model and the real model of the transistor, discrepancies between the actual output circuit losses and EM simulation, and the influence of the chip’s thermal characteristics. Overall, the measured results show a high level of agreement with the simulation results across the entire 2–6 GHz frequency range, indicating excellent chip performance.

Finally, Table 2 summarizes the performance comparison of this power amplifier with other published amplifiers. Compared with the recently reported amplifiers, the chip proposed in this paper almost simultaneously has significantly high efficiency, a rather compact size, and relatively excellent in-band flatness for the same bandwidth and high gain. For instance, it is the most compact in comparison to other published high-gain (greater than 24 dBm) PAs. In addition, it exhibits very good power flatness and gain flatness compared to other high-gain PAs, which are both within ±0.6 dB. In addition, this PA achieves a power-added efficiency range from 39% to 46%, which is fairly good across the whole 2 GHz to 6 GHz band with small efficiency variations.

## 4. Conclusions

A combination of techniques, including distributed equalization, multiplexing the power supply network and matching network, LR dissipative structure, and RC stability network techniques, has been proposed and successfully applied to the implementation of a PA in 0.25 μm GaN HEMT technology. By utilizing this combination technique, the amplifier achieves a very wide flat power frequency response, a high efficiency, a high gain, and a compact size. The PA exhibits a saturated output power of 4 W, power gain of 21 dB, flatness less than ±0.6 dB, and power-added efficiency ranging from 39% to 46% within the whole frequency range of 2–6 GHz. The proposed techniques are expected to be useful methods for wideband power amplifier designs.

## Figures and Tables

**Table 1:** The 6 × 125 m transistor load-pulling data.

Frequency/GHz	Optimal Power Impedance/Ω	Optimal Efficiency Impedance/Ω
2	71.79 + j * 38.32	88.86 + j * 106.07
4	50.50 + j * 40.84	58.27 + j * 83.50
6	36.31 + j * 40.03	32.69 + j * 62.03

**Table 2:** Performance comparison between the proposed PA and other PAs.

Ref.	Freq/GHz	P_sat_/dBm	Gain/dB	PAE/%	Size/mm^2^
[8]	2–6	40.9–41.5	12.8–13.7	27–34	7.6
[9]	2–4	35.1–38.9	11.3–13.4	40–55	3.2
[10]	4–6	33.9–36.1	10–12.2	38–48	2.3
[11]	2–6.5	31.6–33.8	24–27	31.4–51.5	9.6
[13]	2–6	44.4–45.2	NA	35.8–51.3	14.35
[14]	2.4–6	34–36.3	25–35	38–53	3.5 *
[16]	2–6	38.5–40	15	25–30	23
This work	2–6	35.6–36.7	27.2–27.7	39–46	4.95

* External output matching network was not included.

## Data Availability

The data presented in this work are available within the article.
